# Characterizing Effects of Microbial Biostimulants and Whole-Soil Inoculums for Native Plant Revegetation

**DOI:** 10.3390/microorganisms11010055

**Published:** 2022-12-24

**Authors:** Matthew Alfonzetti, Sebastien Doleac, Charlotte H. Mills, Rachael V. Gallagher, Sasha Tetu

**Affiliations:** 1 School of Natural Sciences, Macquarie University, Sydney, NSW 2109, Australia; 2 AirSeed Technologies Australia Pty Ltd., Sydney, NSW 2000, Australia; 3 Hawkesbury Institute for the Environment, Western Sydney University, Locked Bag 1797, Penrith, NSW 2751, Australia; 4 Biomolecular Discovery Research Centre, Macquarie University, Sydney, NSW 2109, Australia; 5 ARC Centre of Excellence in Synthetic Biology, Macquarie University, Sydney, NSW 2109, Australia

**Keywords:** microbial amendment, probiotic, plant growth-promoting bacteria, PGPB, seed enhancement technology, seed coating, extruded pellets, revegetation, soil restoration

## Abstract

Soil microbes play important roles in plant health and ecosystem functioning, however, they can often be disturbed or depleted in degraded lands. During seed-based revegetation of such sites there is often very low germination and seedling establishment success, with recruitment of beneficial microbes to the rhizosphere one potential contributor to this problem. Here we investigated whether Australian native plant species may benefit from planting seed encapsulated within extruded seed pellets amended with one of two microbe-rich products: a commercial vermicast extract biostimulant or a whole-soil inoculum from a healthy reference site of native vegetation. Two manipulative glasshouse trials assessing the performance of two Australian native plant species (*Acacia parramattensis* and *Indigofera australis*) were carried out in both unmodified field-collected soil (trial 1) and in the same soil reduced in nutrients and microbes (trial 2). Seedling emergence and growth were compared between pelleted and bare-seeded controls and analyzed alongside soil nutrient concentrations and culturable microbial community assessments. The addition of microbial amendments maintained, but did not improve upon, high levels of emergence in both plant species relative to unamended pellets. In trial 1, mean time to emergence of *Acacia parramattensis* seedlings was slightly shorter in both amended pellet types relative to the standard pellets, and in trial 2, whole-soil inoculum pellets showed significantly improved growth metrics. This work shows that there is potential for microbial amendments to positively affect native plant emergence and growth, however exact effects are dependent on the type of amendment, the plant species, and the characteristics of the planting site soil.

## 1. Introduction

There is a growing focus on the restoration of terrestrial ecosystems to avoid catastrophic losses of biodiversity, ecosystem functioning, and human livelihoods globally [1,2,3]. However, factors such as seed availability and low establishment rates may restrict the efficacy of restoration efforts [4,5,6], leading to a call for evidence-based science to identify transformative restoration and revegetation advancements [7,8,9]. Microbes play important roles in the germination, growth, health, and stress resilience of plants [10,11] and soil microbial communities have been shown to critically influence soil quality, structure, and nutrient cycling and to act as strong drivers of plant community development [12,13,14,15].

Soil bacteria residing around and on plant root surfaces that are involved in promoting plant growth are termed ‘plant growth-promoting bacteria’ (PGPB), with well characterised examples belonging to diverse genera, including *Azotobacter*, *Bacillus* and *Pseudomonas*. Due to their abilities to promote seed germination and plant growth, performance, and resilience to biotic and abiotic stressors through direct and indirect mechanisms, PGPB thus serve as a potential replacement to chemical fertilizers, pesticides, and other harmful supplements [16,17,18,19]. Benefits to host plants have been shown to be due to roles in breaking down organic matter, increasing inorganic nutrient availability, and the production of phytohormones and compounds that protect against plant pathogens [20]. Mycorrhizal fungi can also help restore low-quality soil and improve plant growth via improving soil water retention and infiltration [21], promoting nutrient availability, and improving soil structure [22,23]. In recognition of these key roles, increasing numbers of bacterial, mycorrhizal and mixed inoculums have been developed commercially for use in agriculture and horticulture [18].

While the majority of research on plant beneficial microbes has focused on agricultural species, there is increasing recognition that manipulation of soil microbial communities to facilitate growth of plant mutualists and improve soil quality may also aid in improving revegetation outcomes in native species [24,25,26,27,28]. In degraded lands, soil microbial communities can be disturbed or depleted [27,29,30]. For example, mining processes [31,32] and common agriculture practices such as tillage [33,34], monoculture plantings [35,36], and the use of fertilizers [37] have been associated with decreases in abundance, diversity, resilience and/or benefits from soil microbes [4]. However, there is the need for more research to determine how best to overcome issues of depauperate soil microbial communities in such sites. 

Microbe-based interventions of potential benefit to restoration have been trialed through a range of techniques. Degraded restoration sites have been inoculated with: whole soil from healthy/intact reference sites [38,39,40]; specific microbial inoculants [41,42,43]; and biostimulants and organic-matter rich additives such as vermicompost [44]. Soil microbial activity has also been enhanced for restoration through indirect actions such as adding woodchips onto the surface of soil [45]. One promising emerging technology is the use of extruded pellets combined with microbial additives. Extruded seed pellets rely on the application of materials such as sand, soils, and clays to seeds using extrusions or molds of slurry or dough-like bulk mixtures to assist in the handling and delivery of seeds [7,46,47]. Such extruded pellets, incorporating additional organic material, trialed by Stock et al. [48] were reported to result in increased soil microbial activity while Madsen et al. [49] suggest extruded pellets incorporating seeds primed with biostimulants, including worm castings (among other additives), show promise for enhancing germination and seedling emergence of perennial grasses. Similarly Pedrini et al. [50] found the application of rhizobia to legumes within seed coatings to improve germination and plant growth while Muller and Berg [51] found the inoculation of oilseed rape seeds with *Serratia plymuthica* via seed pellets was able to contribute to a reduction in Verticillium wilt disease severity. 

Here we investigate the responses of two Australian native plant species to seed encapsulation within extruded seed pellets with and without the incorporation of a commercial vermicast extract biostimulant product or whole-soil inoculum derived from a healthy remnant site, comparing performance to bare seed controls. Two glasshouse trials are reported; the first in which field soil collected from a site flagged for restoration was used as the plant growth substrate, and the second in which a microbially depauperate and nutrient reduced soil mix was used as a growth medium. We characterize nutrient, physicochemical, and microbial community diversity differences between the main growth media provided to all experimental individuals and use this data to aid in interpreting how seed pellet properties may influence growth and emergence responses. Our findings show that application of different mixed microbial inoculums via incorporation in seed pellets can affect native plant seed emergence and growth, indicating potential utility in some restoration scenarios, such as where sites suffer from low soil microbial diversity.

## 2. Materials and Methods

### 2.1. Study Species

Two Australian native plant species were targeted: *Acacia parramattensis* Tindale (Fabaceae; shrub or tree) and *Indigofera australis* Wild. (Fabaceae; shrub). Both species are from the Fabaceae family and produce symbiotic relationships with nitrogen-fixing bacteria in the form of root nodules. Both species are used widely in native plant restoration in eastern Australia. Seed batch viability was confirmed by suppliers.

### 2.2. Growth Trials and Seed Treatments

Two sequential glasshouse trials were established which differed in the potting tube soil media into which seeds were sown. In the setup of both trials, all equipment, soil containers, and potting tubes were washed prior to use with Pyroneg detergent and then disinfected through the application of a 1:49 solution of sodium hypochlorite bleach.

In trial 1, which took place during the austral autumn (February–June), the potting tube soil used was collected a fortnight prior to the establishment of the growth trial from a site at the Australian Botanic Garden (Mount Annan, NSW) (−34.078387, 150.764828; Figure S1) which is undergoing restoration and is underlaid by Wianamatta Group shale. The soil, which was observed to be rich in clay and highly water-retaining, was passed through a 5 mm mesh to homogenize and remove larger organic matter and rocks. In trial 2, which took place during the austral winter (June–September), field soil collected at the same site and using the same method as in trial 1 was homogenized using a sterilized cement mixer with river sand and perlite at a ratio of 5:3:2. Before homogenizing, all three components were placed into autoclave bags (partially filled so that substrate was spread thinly, to a maximum depth of 10 cm) and autoclaved in batches at 121 °C for 30 min at 15 psi, to reduce microbial load, as determined by plate count experiments (described in 2.3). This trial 2 potting-tube mix served as a low nutrient and microbially-depauperate contrast to the unmodified field soil used in trial 1. The following information regarding seed treatments and plant growth conditions applies to both trials unless otherwise specified.

Four seed treatments were tested: an un-enhanced control where seeds were planted directly (‘Bare Seed’); and three types of extruded seed pellet (hereafter ‘Standard Pellet’, ‘Vermicast Extract Pellet’, and ‘Native Soil Pellet’). Immediately prior to direct-planting or pelleting, seeds of *Acacia parramattensis* and *Indigofera australis* were immersed in 90–95 °C water for two minutes to break the hard seed coats of the physically dormant species [52].

The seed pellets (smooth spheres of 1 cm diameter) were formed by hand from approximately 4 g of wet slurry with exactly three seeds of a single species placed at their centroids using sterilized forceps. The full list of ingredients and the proportions at which they were used to make each seed pellet type slurry are presented in Table S1. Standard Pellets were left unamended. Vermicast Extract Pellets incorporated the commercial liquid biostimulant ‘Biocast’ (Island Biologicals Pty Ltd, Oxley Island, Australia; https://www.biocast.com.au (accessed on 28 January 2022)). Containing worm castings, Biocast is advertised to contain a mix of substances including living plant-associated microorganisms (bacteria, fungal spores, and protozoa), plant growth-promoting hormones, enzymes, and fulvic acid. Native Soil Pellets included soil collected from a healthy reference site from a relatively undisturbed area of the Mount Annan Botanic Garden, NSW where the two study species naturally occur (Figure S1). All pellets were left to dry until hard at room temperature for 24 h before planting.

**Figure 1:**
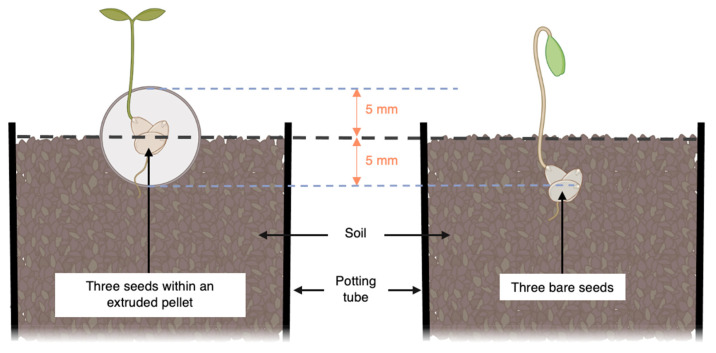
Frontal plane diagram of the upper portion of nursery tubes (6 cm wide sides, 20 cm deep) depicting the depth from the soil surface at which seeds were placed when encapsulated in pellets (**left**; as per seed treatments ‘Standard Pellet’, ‘Vermicast Extract Pellet’, and ‘Native Soil Pellet’) and when kept bare (**right**; as per seed treatment ‘No Pellet’). Note that the relative sowing depths of the bare and the pelleted seeds are equal (c. 5 mm). Figure created using elements from BioRender.com (accessed on 15 September 2022).

Each combination of species and seed treatment was set up with 26 separate potting tube replicates (20 cm tall by 6 cm wide square potting tubes filled with the unmodified field soil in trial 1 and the autoclaved soil mix in trial 2). Each potting tube received a single seed pellet which was placed so that they were half covered by the potting tube soil (Figure 1). The bare seeds (exactly three of a single species) were placed at a depth of 0.5 cm from the soil surface so that all seeds (bare or pelleted) were at the same relative sowing depth (Figure 1). 

Potting tubes were placed within 20-position trays segregated by seed treatment designations to avoid cross-contamination of materials and microbes. Potting tube trays were randomly positioned within a climate-controlled glasshouse and were re-randomized every seven days to promote even exposure to any varying conditions within the glasshouse (e.g., sun angles and shadows, air-conditioning currents, sprinkler outputs, etc.). Glasshouse temperatures were set to a 25 °C day and 19 °C night 12 h cycle. Automated ceiling mounted sprinklers were programmed to open for one minute at 8:00 am and 2:00 pm daily for the duration of the experiment.

### 2.3. Nutrient and Physicochemical Analyses Microbial Community Diversity Quantifications

Nutrient and physicochemical analyses were performed at the Environmental Analysis Laboratory, Southern Cross University on triplicate 50 mL samples of each potting tube soil type, and triplicate 50 mL samples of the freshly made Standard Pellet slurry mixture. The samples were sieved and lightly crushed to <2 mm. Analyses were performed following standard methods described in Rayment & Lyons [53] apart from total phosphorous and total nitrogen, which were analyzed via an in-house LECO Trumac Analyzer method. At the start of every batch three blanks and a reference sample were run as well as a single blank and reference sample every 40 samples. Standard reference material were run alongside and results compared against the reference and blank corrected.

### 2.4. Microbial Community Diversity Quantifications

The culturable microbial diversity of both field soils from the restoration site and the healthy native reference site at Mount Annan and the autoclaved soil mix was investigated via culturing on tryptic soy agar plates (Edwards Group Holdings, Narellan, Australia). Three samples of each soil type were serially diluted in sterile phosphate buffer-saline (Sigma-Aldrich, Missouri, United States) to concentrations of 1:1000, 1:2000, 1:4000, 1:8000, 1:16,000 and 1:32,000 (*m*/*v*). All dilutions and a buffer only control were plated in triplicate and incubated at 25 °C for five days. Only the 1:16,000 and 1:32,000 dilutions were plated for the Mount Annan field soil samples while for the autoclaved soil mix all dilutions were plated to account for the anticipated diminished microbial diversity and richness after autoclaving. Morphotypes were categorized according to their visual characteristics (form, elevation, margin, surface, opacity, and pigmentation).

### 2.5. Seedling Emergence, Survival, and Growth Data Collection

Seedling emergence (scored as a seedling successfully penetrating through the soil) was monitored every Monday, Wednesday and Friday after planting for ten weeks in trial 1 and twelve weeks in trial 2. To standardize growth conditions within each replicate, after one seedling had emerged, other emergents were removed immediately upon detection. Throughout the trial, plants were also monitored for evidence of disease or damage and the date of any deaths and their speculated or known causes recorded.

Plants were harvested eight weeks from their individual emergence dates in weekly batches. Potting tubes with plants due for harvest were removed from the glasshouses and their contents carefully excised. Excised plants were cut at the root–shoot boundary and, after the roots were gently washed free of soil in warm water, root nodules were individually removed with forceps and counted. Biomass (mg) and root:shoot ratios (excluding nodules) were determined by weighing plant material dried at 70 °C for ≥72 h. All weights were measured using an analytical balance (Mettler Toledo, Port Melbourne, VIC, Australia).

### 2.6. Statistical Analyses

All statistical analyses and figures were generated in R version 4.0.0 [54] using the packages: *stats*, *ggplot2* [55], *car* [56], *FactoMineR* [57], *emmeans* [58], and *lme4* [59]. Data were tested for normality and homogeneity of variance and were transformed when appropriate. All statistical outputs were considered significant at an alpha value of 0.05.

Principal component analysis (PCA) was employed to summarize and visualize differences in nutrient and physicochemical parameters among the two potting tube soil types and the Standard Pellet slurry mixture. The principal components were calculated from the full set of scaled (standardized) data. As the first two principal components collectively captured a high amount of the variation in the data (93.1%), they were used to construct a PCA biplot. 

The number of morphotypes and CFU per mL of each of the soil and seed pellet samples were analyzed using Ordinary Least-Squares (OLS) regression including the sample type as an independent variable. One-way ANOVAs were employed to determine significant differences and multiple comparisons of means were analyzed using Tukey’s multiple comparisons adjustment.

Emergence counts, mean time to emergence (average lag time between seed imbibition and seedling emergence in days), survival, and plant growth data were analyzed separately for each growth trial and species and all models included the independent fixed variable ‘seed treatment’. Emergence counts were fit to Generalized Linear Models (GLMs) and emergence times were fit to Residual Maximum Likelihood (REML) linear models; both models also included a random effect (potting tube ID) to account for the three seeds in each potting tube. Seedling survival at the end of each growth trial was also fit using GLMs. The data related to plant growth—namely total plant biomass, root:shoot ratios, and total nodule counts—were fit using OLS regression. Models were analyzed through one-way ANOVAs and multiple comparisons of means were analyzed using Tukey’s multiple comparisons adjustment.

## 3. Results

Here we investigated the emergence, survival, and growth responses of two Australian native plant species grown from extruded pellets amended with either a microbial biostimulant or whole-soil inoculum in two glasshouse trials using (1) unmodified field-collected soil, and (2) a microbially-depauperate and nutrient-reduced soil-sand-perlite mix. In each trial, both unamended pellets and bare-seeded controls were run alongside each treatment for comparison. Results were analyzed alongside quantifications of soil nutrient/physicochemical parameters and culture-based microbial community diversity assessments.

### 3.1. Nutrient and Physicochemical Properties of Extruded Seed Pellets and Soils

**Figure 2:**
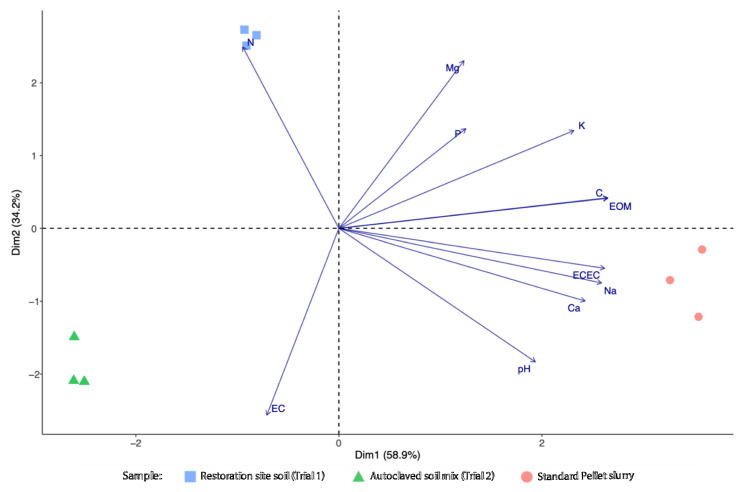
Principal Component Analysis biplot of the nutrient and physicochemical properties of samples from the two soil types and the mixture used to make the standard extruded seed pellets. Symbols represent the scores of triplicate samples; vectors represent the loadings of variables (nutrient/physicochemical parameters). C: total carbon; Ca: exchangeable calcium; EC: electrical conductivity; ECEC: effective cation exchange capacity; EOM: estimated organic matter; K: exchangeable potassium; Mg: exchangeable magnesium; N: total nitrogen; Na: exchangeable sodium; P: phosphorus.

Nutrient and physicochemical parameter quantifications of the restoration site soil (trial 1), the autoclaved soil mix (trial 2), and the extruded seed pellet slurry used to make the Standard Pellets are reported in Table 1 and summarized in the PCA (Figure 2). The first principal component, explaining 58.9% of the variation, is strongly correlated with concentrations of organic matter, total carbon, exchangeable nutrients (calcium, potassium, and sodium), effective cation exchange capacity (ECEC) and pH. The second principal component, explaining 34.2% of the variation, was positively correlated with total nitrogen and magnesium, and was negatively correlated with electrical conductivity. Relative to the two soil types, the standard pellet mixture was shown to be richer in organic matter and most elements (particularly total carbon, calcium, potassium, and sodium), as well as having a higher pH. Both field soils had higher levels of organic matter, total carbon and nitrogen, exchangeable magnesium and potassium, but lower electrical conductivity than the autoclaved soil mix, demonstrating the efficacy of this treatment in lowering nutrient availability. 

### 3.2. Microbial Community Diversity

The healthy reference site soil, used as an inoculum, contained the highest load of culturable microbes, containing an average of 1.2 × 10^5^ ± 1.6 × 10^4^ (SE) CFU per gram of field soil. In comparison, the trial 1 planting soil collected at the restoration site contained 6.3 × 10^4^ ± 9.5 × 10^3^ (SE) CFU per gram, while the autoclaved soil mix used in trial 2 contained an average of 1.3 × 10^4^ ± 2.3 × 10^3^ (SE) CFU per gram. Significant differences occurred between these sample types (F_2,83_ = 30.19, *p* < 0.0001) with values being significantly lower in the autoclaved soil mix compared with both the restoration site field soil (*p* < 0.0001) and the native reference site field soil (healthy soil inoculum) (*p* < 0.0001).

The number of unique microbial morphotypes identified in the healthy reference site soil used as an inoculum and the trial 1 planting soil was similar, with 17 and 20 morphotypes identified respectively (Figure S2). In comparison, only four morphotypes were differentiated within the autoclaved soil mix (trial 2).

### 3.3. Seedling Emergence

**Figure 3:**
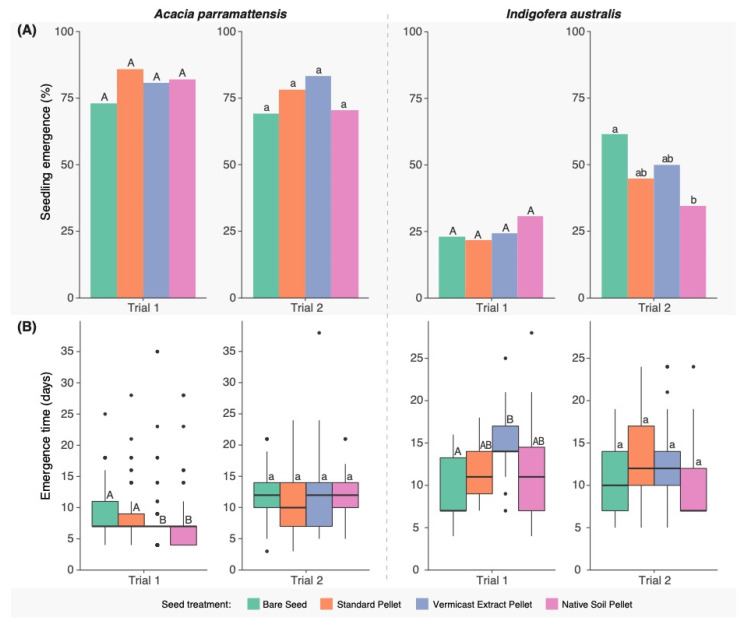
(**A**) Percentage of seeds of two native Australian plant species that emerged, and (**B**) time to emergence (days), from three pelleting treatments and bare seed controls. Emergence precents and mean emergence times without shared letters differ at *p* < 0.05 (ANOVA and Tukey test) within species and trials (upper- vs. lower-case letters).

Emergence was consistently high for *A. parramattensis* across both trials, with no significant differences detected in emergence (%) between any seed treatments (Figure 3a). In *I. australis* however, marginally significant differences were detected in trial 2 (χ^2^_3_ = 7.86, *p* = 0.049) where emergence was significantly lower by 27% in the Native Soil Pellets compared with the Bare Seeds (*p* = 0.03).

During trial 1, significant differences in mean time to emergence (days) were detected between seed treatments in both species (*A. parramattensis*: F_3,100_ = 13.20, *p* < 0.001; *I. australis*: F_3,48_ = 3.68, *p* = 0.02), but not in trial 2 (Figure 3b). During trial 1, time to emergence of *A. parramattensis* was significantly reduced in both amended pellet types (Native Soil and Vermicast Extract) relative to Bare Seed and Standard Pellet controls (Vermicast Extract Pellet-Bare Seed: *p* < 0.01; Vermicast Extract Pellet-Standard Pellet: *p* = 0.03; Native Soil Pellet-Bare Seed: *p* < 0.0001; Native Soil Pellet-Standard Pellet: *p* < 0.0001), however, emergence of *I. australis* was significantly slower in the Vermicast Extract Pellets relative to the Bare Seed control (*p* = 0.01).

### 3.4. Seedling Survival

Percent survival of seedlings that emerged was high throughout both growth trials and, at the time of harvest, no significant differences were observed between seed treatments in either species (Tables S3 and S4).

### 3.5. Post-Harvest Plant Growth Metrics

**Figure 4:**
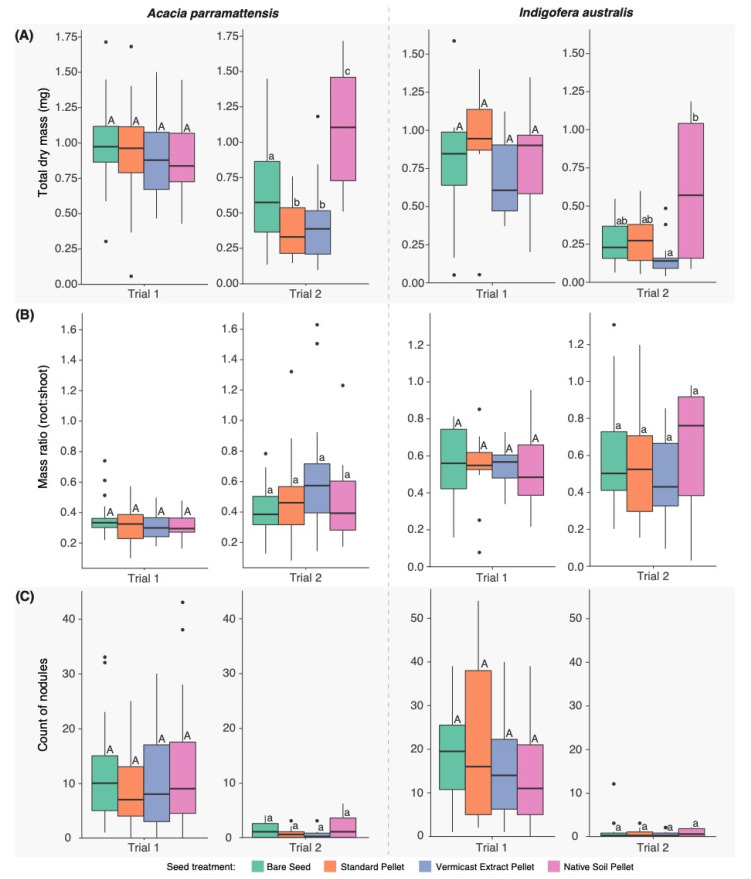
(**A**) Total biomass (mg) of plants, (**B**) ratios of root dry weight (mg) to shoot dry weight (mg), and (**C**) count of nodules per plant at harvest in four seed treatments. Means without shared letters differ at *p* < 0.05 (ANOVA and Tukey test) within species and trials (upper- vs. lower-case letters).

Total dry biomass (mg) of seedlings at harvest (Figure 4a) was not significantly different between seed treatments in trial 1. However, in trial 2, significant differences occurred in both *A. parramattensis* (F_3,97_ = 21.08, *p* < 0.001) and *I. australis* (F_3,59_ = 5.57, *p* < 0.01). In *A. parramattensis*, total dry biomass was significantly higher in the Native Soil Pellets compared to all other seed treatments (Bare Seeds: *p* < 0.01; Standard Pellets: *p* < 0.001; Vermicast Extract Pellets: *p* < 0.001), however, was significantly lower in the two other extruded pellet types compared with the Bare Seeds (Standard Pellets: *p* = 0.02; Vermicast Extract Pellets: *p* = 0.01). In *I. australis*, the only significant differences detected were between the two types of amended pellets whereby total dry biomass was higher in the Native Soil Pellets compared with the Vermicast Extract Pellets (*p* < 0.001).

There were no significant differences between seed treatments in the root:shoot mass ratios nor the counts of root nodules in either species in either of the trials (Figure 4b,c).

## 4. Discussion

Soil microbes play important roles in shaping plant health and ecosystem function, and soil organisms and processes are increasingly being incorporated into restoration projects [24,25,26,27,28,60,61]. A range of commercial microbial products exist, although they are generally developed and marketed for agricultural applications, and as such, most of the related research also focuses on their application to cropping species (e.g., VESTA, SOBEC Corporation, Fowler, CA; [62]). However, there is growing evidence that extruded seed pellets designed to supply beneficial native microbes [63] or otherwise support favorable microbiomes [48] may be beneficial in the restoration of highly disturbed soils such as post-mining tailings where native microbial communities and nutrient profiles are often altered and seedling establishment and growth are below target levels [26,31,64].

Here, using two glasshouse trials, we demonstrate how extruded seed pellets maintain high seedling emergence and survival in two commonly-used restoration target species, and that the growth of these species may be improved through the incorporation of a native whole-soil inoculum from a healthy reference site. However, limited evidence of benefits from the addition of a vermicast extract product into the pellets was found, and we identify a few cases where, relative to bare-seeded controls, the vermicast amendment resulted in reduced plant performance. Past research has shown that in some situations worm castings can significantly restore soil health and increase plant growth by promoting nutrient uptake and resistance to pathogens [65]. However, positive effects from worm castings may depend on doses and higher doses may improve seedling quality but at the expense of germination [66]. Vermicast delivered as soil pre-treatment or post-treatments instead of inoculum in extruded seed pellets may result in better outcomes for germination and plant development. Further testing, involving different concentrations or application methods, would assist in determining where such products may provide benefit.

Cultivable microbe abundance and diversity was similar between trial 1′s potting tube soil collected from the Mount Annan field restoration site and the healthy reference soil added as an inoculum to the Native Soil Pellets. This may explain why the addition of either amendment type into the extruded pellets failed to promote seedling emergence proportions and growth in trial 1. Any potential benefits from plant-growth-promoting bacteria and fungi from the amended pellets may have been diminished due to there being very few limiting-factors to plant growth including accessible nutrients and relevant native microbes which were already likely present in the potting tube soil—an issue proposed in a previous study of similar extruded seed pellets amended with a commercial soil probiotic [67]. Following this, trial 2 was designed to test the amended pellets in a growth substrate that was lower in nutrients and microbially-depauperate.

In trial 2 growth of *A. parramattensis* and *I. australis* was greater in the Native Soil Pellets than in all other treatments, however in the latter species the difference was only statistically significant compared with Vermicast Extract Pellets. The plant species specific differences in response to microbial inoculums seen here are consistent with what has been reported previously. For example, Dadzie et al. [68] found that the benefit of cyanobacteria or native heterotrophic bacteria on plant establishment from extruded pellets was species specific and the former inoculation resulted in a 11% reduction in growth of *Triodia epactia* (Poaceae) seedlings. Similarly, the application of commercially-available soil microbial amendments in semi-arid ecosystems has been shown have varying effects on plant fitness, possibly as a result of plant-microbiome physiological mismatching as well as issues of low persistence of consortium members [25].

Compared with commercial products, whole-soil applications from healthy remnant areas have been suggested to have greater old-field restoration effects with benefits that could last for several years [69]. However, the application of native soil inoculums in restoration work also has some potential risks such as disturbance of the donor sites’ soil, seed bank, and biota, and the introduction of plant pathogens, such as phytophthora, into previously un-infected areas. To avoid this, pathogen screening may be required prior to use in any field setting. Furthermore, the use of some combinations of bacteria in concert have been shown to result in unexpected diminished benefits compared with the use of single-strain inoculants [70,71,72,73]. Further testing, considering both single strain and mixed microbe inoculants may help provide useful information towards informed selection of appropriate microbes to be applied for restoration of specific native plant species.

Both plant species trialed here are known to form symbiotic relationships with specific nitrogen-fixing bacteria, resulting in the formation of root nodules. Counts of root-nodules were substantially lower in trial 2 compared to trial 1, indicating access to appropriate nodule-forming bacteria was limited, consistent with this second growth substrate being microbially-depauperate. Interestingly however, neither of the microbial amendments ameliorated this, indicating that they did not provide the requisite bacteria to restore normal nodule development. Previous work with commercial microbial products, testing a rhizobia product and a mixed bacteria-and-fungi product on pigeon pea has shown that in some situations they can successfully increase the number of root-nodules, although in this work the inoculums did not improve measured plant-growth parameters relative to control treatments [74], while another study on pigeon pea identified three non-rhizobial isolates that promote nodule number and fresh weigh as well as plant growth, but only when co-inoculated with the rhizobial bioinoculant strain IC3123 [75]. It is also worth considering that evidence from previous studies indicates that the incorporation of targeted microbes into extruded pellets might be challenging, with microbe viability potentially declining due to osmotic stress [76] or desiccation [77] during the drying of the freshly-formed pellets. It is possible that nodule forming genera present within the two tested amendments may have been susceptible to these stressors and were therefore lost or not able to successfully colonize the host roots.

This study did not extend to exploring the taxonomic identity and relative abundance of microbial communities within the microbial amendments nor at the root-soil interface for seedlings from different treatments, however, long-term microbial persistence and community shifts associated with different amendment additions mark particularly important areas for further research. This may also be especially important in the clarification of where any benefits/disadvantages to plant fitness are imparted by the amendments and in distinguishing if these effects are indeed microbially derived or imparted by abiotic factors. It should also be acknowledged that trials were conducted under near-optimal growth conditions, and that additional benefits of microbial amendments may be identified in studies where plants are subject to one or more stressors. Field trials in degraded soils are considered imperative in future quantifications of the effects of microbial amendments in extruded seed pellets for native species revegetation.

## 5. Conclusions

In response to the restoration challenges which stem from the current biodiversity crisis, the development of new, transformative, and efficient mass-scale revegetation techniques is critical to avoid catastrophic losses of ecosystem function. A growing corpus of literature recognizes the importance of considering soil mutualists for native species revegetation. Although the potential for microbial amendments to positively affect native plant emergence and growth has been demonstrated in some contexts, often the effects are species-specific, and can be complicated by the characteristics of the planting site soil or when used in concert with other technologies such as extruded pellets. The findings of this paper further indicate the necessity for collaboration between restoration practitioners, microbiologists, and ecologists to find suitable scalable solutions. Further research into the specific microbial communities of Australian soils, the development of standardized methods for selecting and applying microbial amendments will be useful, along with considerations of how the effects of amendment vary over a broader floral diversity, and careful monitoring of the impacts on native microbial communities.

## Figures and Tables

**Table 1:** Nutrient and physicochemical properties (mean ± SE) of the soil collected at the restoration site at Mount Annan (Trial 1), the autoclaved soil mix (Trial 2), and the standard extruded seed pellet slurry used to form the Standard Pellets. All results presented as a 40 °C oven dried weight. For all sample types, *n* = 3.

Parameter	Restoration Site Soil	Autoclaved Soil Mix	Standard Pellet Slurry
Estimated organic matter (%OM)	9.36 ± 0.29	4.03 ± 0.09	16.79 ± 0.80
Total carbon (%)	5.36 ± 0.18	2.30 ± 0.06	9.59 ± 0.46
Exchangeable magnesium (mg/kg)	413.33 ± 2.73	207.00 ± 4.16	347.14 ± 16.17
Exchangeable calcium (mg/kg)	2562.67 ± 58.87	2708.00 ± 103.91	4167.55 ± 238.95
Exchangeable potassium (mg/kg)	556.00 ± 5.20	268.33 ± 4.67	656.21 ± 22.27
Exchangeable sodium (mg/kg)	47.67 ± 13.68	61.00 ± 1.00	1840.53 ± 26.34
Phosphorus (mg/kg P)	6.47 ± 0.12	4.00 ± 0.06	6.57 ± 1.79
Total nitrogen (%)	0.36 ± 0.01	0.12 ± 0.00	0.09 ± 0.01
pH	6.80 ± 0.00	7.60 ± 0.00	8.50 ± 0.05
Electrical Conductivity (dS/m)	0.16 ± 0.00	0.88 ± 0.07	0.52 ± 0.02
Effective Cation Exchange Capacity (cmol + /kg)	17.67 ± 0.33	16.00 ± 0.58	33.34 ± 1.39

## Data Availability

Not applicable.

## Supplementary Materials

The following supporting information can be downloaded at: https://www.mdpi.com/article/10.3390/microorganisms11010055/s1, Figure S1: Field soil collection sites; Table S1: Extruded seed pellet component proportions; Figure S2: Representative tryptic soy agar microbial plates; Table S2: Percentage of emerged seedlings that remain alive per fortnight; Table S3: statistical comparisons of seedling survival percentages at harvest.
