# Supplementary material for: Characterizing Effects of Microbial Biostimulants and Whole-Soil Inoculums for Native Plant Revegetation
Source: Microorganisms. 2022 Dec 24;11(1):55. doi: 10.3390/microorganisms11010055 (PMC9867050; doi:10.3390/microorganisms11010055)
Supplement: Supplementary file 1 [file microorganisms-11-00055-s001.zip › microorganisms-2114757-supplementary.pdf]

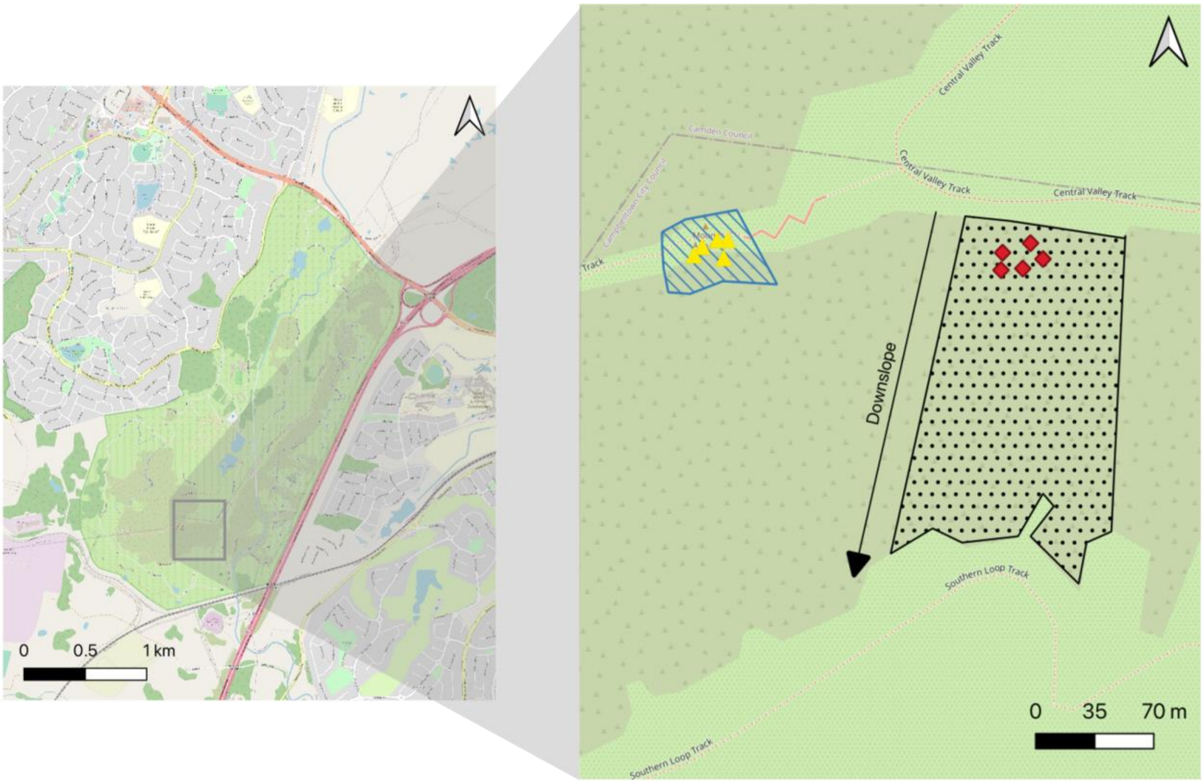

Figure S1: Soil Collection Sites at Mount Annan, Australian Royal Botanic Gardens, Sydney NSW underlaid by Wianamatta Group shale (-34.078387, 150.764828). The dot-filled area is targeted for restoration from which the soil used in the first trial was collected. The line-filled area is remnant of Western Sydney dry sclerophyll rainforest where the two study species naturally occur and thus is used as the healthy reference site from which the whole soil inoculum was sourced. At each location, the soil top layer was collected from five randomly selected areas.

Table S1: Proportions of each component of the extruded seed pellet mixtures per treatment. All components, including liquids, where weighed in grams.

Component	Standard Pellet	Vermicast Extract Pellet	Native Soil Pellet
Cardboard pulp	4.59%	4.48%	2.3%
Clay	1.53%	1.49%	1.53%
Zeolite	15.01%	14.63%	15.01%
Sand	20.37%	19.85%	20.37%
Activated charcoal	6.43%	6.27%	6.43%
Water	52.07%	50.75%	52.07%
Vermicast extract	-	2.54%	-
Native Soil Inoculum	-	-	2.3%

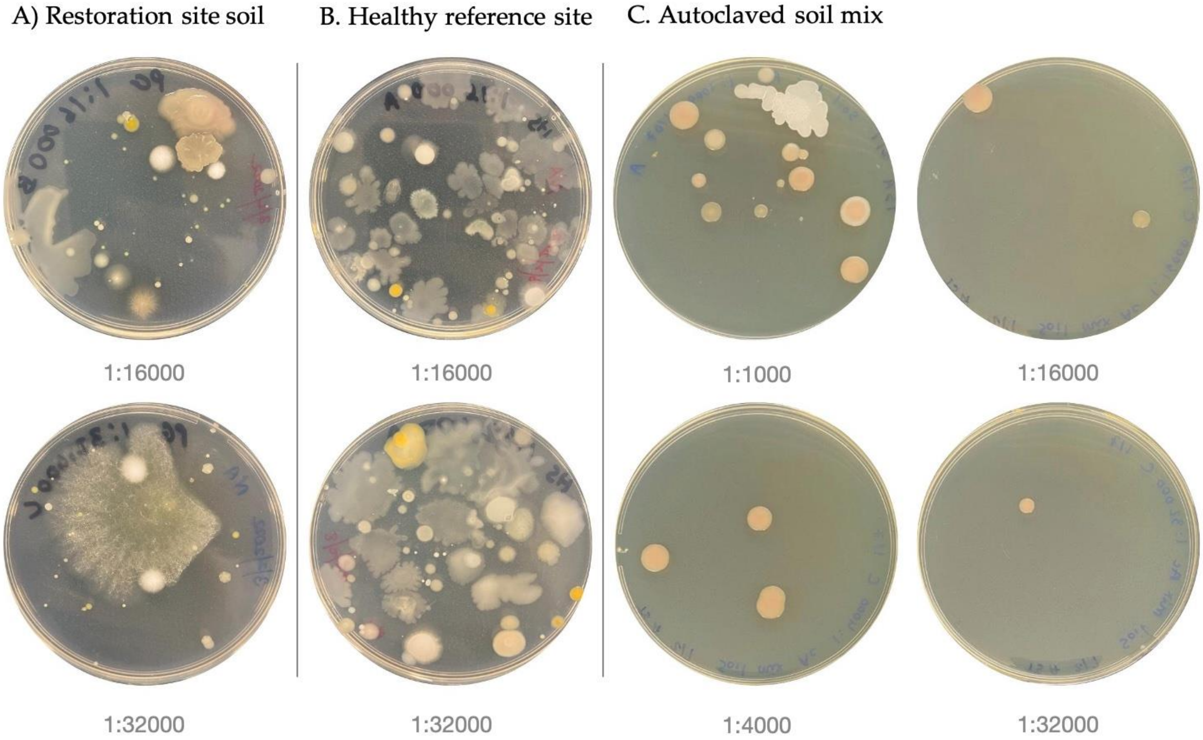

Figure S2: Representative tryptic soy agar microbial plates colonies of cultivable microbes observed in A) soil collected at the restoration site at Mount Annan (Trial 1), B) the healthy reference site, and C) the autoclaved soil mix (Trial 2) at multiple PBS dilutions (m/v). Samples were taken at the time of planting.

Table S2: Percentage of emerged seedlings that remain alive (%) per fortnight for each species, trial, and seed treatment.

Species	Trial	Treatment	Seedling survival at the end of each fortnight (%)				
			**0-14**	**14-28**	**28-42**	**42-56**	**56-70**
Acacia parramattensis	Trial 1	Bare Seed	100	100	100	96.15	96.15
		Standard Pellet	100	100	100	96.15	96.15
		Vermicast Extract Pellet	100	100	100	96.15	96.15
		Native Soil Pellet	96.15	96.15	96.15	92.31	92.31
	Trial 2	Bare Seed	100	100	100	100	100
		Standard Pellet	100	100	100	100	100
		Vermicast Extract Pellet	100	100	100	100	100

		Native Soil Pellet	96	96	96	96	96
Indigofera australis	Trial 1	Bare Seed	100	100	100	100	100
		Standard Pellet	100	100	100	100	100
		Vermicast Extract Pellet	100	90.91	90.91	90.91	90.91
		Native Soil Pellet	93.75	93.75	93.75	93.75	93.75
	Trial 2	Bare Seed	100	100	100	100	100
		Standard Pellet	100	100	100	95	95
		Vermicast Extract Pellet	100	94.74	84.21	84.21	84.21
		Native Soil Pellet	88.24	82.35	82.35	82.35	76.47

Table S3: Statistical comparisons of percentage of emerged seedlings that remain alive (%) at the end of the growth trials. One-way ANOVAs were utilized and, where appropriate, multiple comparisons of means were analyzed using Tukey's multiple comparisons adjustment. Separate comparisons were made between seed treatments per species and trials.

**Species**	**Trial**	**One-way ANOVAs output**	**Post-hoc output**
Acacia parramattensis	Trial 1	$\chi^2_3 = 2.77$, $p = 0.42$	-
	Trial 2	$\chi^2_3 = 8.30$, $p = 0.0401$	All pairwise comparisons: p > 0.1
Indigofera australis	Trial 1	$\chi^2_3 = 0.57$, $p = 0.90$	-
	Trial 2	$\chi^2_3 = 2.84$, $p = 0.42$	-
